# Supplementary material for: HIVprotI: an integrated web based platform for prediction and design of HIV proteins inhibitors
Source: J Cheminform. 2018 Mar 9;10:12. doi: 10.1186/s13321-018-0266-y (PMC5845081; doi:10.1186/s13321-018-0266-y)
Supplement: Supplementary file 2 — Additional file 2. Source code of HIVProtI web server. [file 13321_2018_266_MOESM2_ESM.zip › HIVprotI_Source-code/hivprotI/weka-3-6-6/doc/weka/classifiers/bayes/net/VaryNode.html]

VaryNode


---


|  |  |  |
| --- | --- | --- |
| **Overview** \| **Package** \| **Class** \| **Tree** \| **Deprecated** \| **Index** \| **Help** \| **Weka's home** | |  |
| **PREV CLASS**   NEXT CLASS | **FRAMES**    **NO FRAMES**     **All Classes** |  |
| SUMMARY: NESTED \| FIELD \| CONSTR \| METHOD | DETAIL: FIELD \| CONSTR \| METHOD |  |


---


## weka.classifiers.bayes.net Class VaryNode

```
java.lang.Object
  weka.classifiers.bayes.net.VaryNode
```

**All Implemented Interfaces:**: java.io.Serializable, RevisionHandler

---

``` public class VaryNode extends java.lang.Object implements java.io.Serializable, RevisionHandler ```

Part of ADTree implementation. See ADNode.java for more details.

**Version:**
:   $Revision: 1.6 $

**Author:**
:   Remco Bouckaert (rrb@xm.co.nz)

**See Also:**: Serialized Form

---

| **Field Summary** | |
| --- | --- |
| `ADNode[]` | `m_ADNodes`             list of ADNode children |
| `int` | `m_iNode`             index of the node varied |
| `int` | `m_nMCV`             most common value |


**Constructor Summary**

`VaryNode(int iNode)`
  
          Creates new VaryNode


| **Method Summary** | |
| --- | --- |
| `void` | `getCounts(int[] nCounts, int[] nNodes, int[] nOffsets, int iNode, int iOffset, ADNode parent, boolean bSubstract)`             get counts for specific instantiation of a set of nodes |
| `java.lang.String` | `getRevision()`             Returns the revision string. |
| `void` | `print(java.lang.String sTab)`             print is used for debugging only, called from ADNode |

**Methods inherited from class java.lang.Object**

`equals, getClass, hashCode, notify, notifyAll, toString, wait, wait, wait`

**Field Detail**

### m\_iNode

```
public int m_iNode
```

:   index of the node varied

---


### m\_nMCV

```
public int m_nMCV
```

:   most common value

---


### m\_ADNodes

```
public ADNode[] m_ADNodes
```

:   list of ADNode children


**Constructor Detail**

### VaryNode

```
public VaryNode(int iNode)
```

:   Creates new VaryNode


**Method Detail**

### getCounts

```
public void getCounts(int[] nCounts,
                      int[] nNodes,
                      int[] nOffsets,
                      int iNode,
                      int iOffset,
                      ADNode parent,
                      boolean bSubstract)
```

:   get counts for specific instantiation of a set of nodes

    :   **Parameters:**: `nCounts` - array for storing counts: `nNodes` - array of node indexes: `nOffsets` - offset for nodes in nNodes in nCounts: `iNode` - index into nNode indicating current node: `iOffset` - Offset into nCounts due to nodes below iNode: `parent` - parant ADNode of this VaryNode: `bSubstract` - indicate whether counts should be added or substracted

---


### print

```
public void print(java.lang.String sTab)
```

:   print is used for debugging only, called from ADNode

    :   **Parameters:**: `sTab` - amount of space.

---


### getRevision

```
public java.lang.String getRevision()
```

:   Returns the revision string.

    :   **Specified by:**: `getRevision` in interface `RevisionHandler`
    :   **Returns:**: the revision


---


|  |  |  |
| --- | --- | --- |
| **Overview** \| **Package** \| **Class** \| **Tree** \| **Deprecated** \| **Index** \| **Help** \| **Weka's home** | |  |
| **PREV CLASS**   NEXT CLASS | **FRAMES**    **NO FRAMES**     **All Classes** |  |
| SUMMARY: NESTED \| FIELD \| CONSTR \| METHOD | DETAIL: FIELD \| CONSTR \| METHOD |  |


---
